# Attenuation by all-*trans*-retinoic acid of sodium chloride-enhanced gastric carcinogenesis induced by *N*-methyl-*N***′**-nitro-*N*-nitrosoguanidine in Wistar rats

**DOI:** 10.1038/sj.bjc.6690117

**Published:** 1999-02

**Authors:** M Tatsuta, H Iishi, M Baba, R Hirasawa, H Yano, N Sakai, A Nakaizumi

**Affiliations:** Department of Gastrointestinal Oncology, Osaka Medical Center for Cancer and Cardiovascular Diseases, Osaka, 537-8511, Japan

**Keywords:** all-*trans*-retinoic acid, sodium chloride, gastric carcinogenesis, transforming growth factor α, apoptosis

## Abstract

The effect of prolonged administration of all-*trans*-retinoic acid (RA) on sodium chloride-enhanced gastric carcinogenesis induced by *N*-methyl-*N*′-nitro-*N*-nitrosoguanidine, and the labelling and apoptotic indices and immunoreactivity of transforming growth factor (TGF) α in the gastric cancers was investigated in Wistar rats. After 25 weeks of carcinogen treatment, the rats were given chow pellets containing 10% sodium chloride and subcutaneous injections of RA at doses of 0.75 or 1.5 mg kg^−1^ body weight every other day. In week 52, oral supplementation with sodium chloride significantly increased the incidence of gastric cancers compared with the untreated controls. Long-term administration of RA at both doses significantly reduced the incidence of gastric cancers, which was enhanced by oral administration of sodium chloride. RA at both doses significantly decreased the labelling index and TGF-α immunoreactivity of gastric cancers, which were enhanced by administration of sodium chloride, and significantly increased the apoptotic index of cancers, which was lowered by administration of sodium chloride. These findings suggest that RA attenuates gastric carcinogenesis, enhanced by sodium chloride, by increasing apoptosis, decreasing DNA synthesis, and reducing TGF-α expression in gastric cancers. © 1999 Cancer Research Campaign


*British Journal of Cancer* (1999) **79**(5/6), 732–736
© 1999 Cancer Research Campaign
Article no. bjoc.1998.0117

### Attenuation by all-*trans*-retinoic acid of sodium chloride-enhanced gastric carcinogenesis induced by *N*-methyl-*N*′-nitro-*N*-nitrosoguanidine in Wistar rats

**M Tatsuta, H Iishi, M Baba, R Hirasawa, H Yano, N Sakai and A Nakaizumi**

Department of Gastrointestinal Oncology, Osaka Medical Center for Cancer and Cardiovascular Diseases, Osaka 537-8511, Japan

**Summary** The effect of prolonged administration of all-*trans*-retinoic acid (RA) on sodium chloride-enhanced gastric carcinogenesis induced by *N*-methyl-*N*′-nitro-*N*-nitrosoguanidine, and the labelling and apoptotic indices and immunoreactivity of transforming growth factor (TGF) α in the gastric cancers was investigated in Wistar rats. After 25 weeks of carcinogen treatment, the rats were given chow pellets containing 10% sodium chloride and subcutaneous injections of RA at doses of 0.75 or 1.5 mg $kg^{-1}$ body weight every other day. In week 52, oral supplementation with sodium chloride significantly increased the incidence of gastric cancers compared with the untreated controls. Long-term administration of RA at both doses significantly reduced the incidence of gastric cancers, which was enhanced by oral administration of sodium chloride. RA at both doses significantly decreased the labelling index and TGF-α immunoreactivity of gastric cancers, which were enhanced by administration of sodium chloride, and significantly increased the apoptotic index of cancers, which was lowered by administration of sodium chloride. These findings suggest that RA attenuates gastric carcinogenesis, enhanced by sodium chloride, by increasing apoptosis, decreasing DNA synthesis, and reducing TGF-α expression in gastric cancers.

**Keywords:** all-*trans*-retinoic acid; sodium chloride; gastric carcinogenesis; transforming growth factor α; apoptosis

Sodium chloride is closely linked to the development of experimental gastric cancers in the initiation and promotion stages (Shirai et al, 1984; Takahashi et al, 1984; Tatsuta et al, 1995). We recently found that dietary high protein significantly attenuates sodium chloride-enhanced gastric carcinogenesis in Wistar rats (Tatsuta et al, 1997). Retinoids are a family of natural or synthetic compounds structurally related to vitamin A (Toma et al, 1997) that have been shown to induce apoptosis in various cell lines (Delia et al, 1993; Ponzoni et al, 1995). These facts suggest that all-*trans*-retinoic acid (RA) might attenuate sodium chloride-enhanced gastric carcinogenesis.

The carcinogen *N*-methyl-*N*′-nitro-*N*-nitrosoguanidine (MNNG) caused a significant increase in the intensity of transforming growth factor (TGF) α expression in the gastric mucosa (Livingstone et al, 1994). Recently, we found that prolonged administration of TGF (34–43)-α, an antagonist of TGF-α, significantly reduced the incidence of gastric cancers induced by MNNG (Tatsuta et al, 1998). Therefore, to investigate this possibility, we examined the effects of RA on gastric carcinogenesis induced by MNNG and on the TGF-α expression of gastric cancers in Wistar rats.

### MATERIALS AND METHODS

### Animals

One hundred and twenty young (6-week-old) inbred male Wistar rats purchased from Japan SLC (Shizuoka, Japan) were housed in suspended, wire-bottomed cages at controlled temperature (21–22°C) and humidity (30–50%), with a 12 h–12 h light–dark cycle.

*Received 6 April 1998*
*Revised 13 July 1998*
*Accepted 16 July 1998*

*Correspondence to:* M Tatsuta, Department of Gastrointestinal Oncology, Osaka Medical Center for Cancer and Cardiovascular Diseases, 3-3, Nakamichi 1-chome, Higashinari-ku, Osaka 537-8511, Japan

### Carcinogen and treatment

The rats were given drinking water containing MNNG (25 μg $ml^{-1}$; Aldrich Chemical, Milwaukee, WI, USA) and regular chow pellets (Nihon Nosan, Yokohama, Japan) for 25 weeks. The MNNG was dissolved in deionized water at a concentration of 1 mg $ml^{-1}$ and kept in a cool, dark place. The stock solution was diluted to 25 μg $ml^{-1}$ with tap water just before use. Each rat was given the MNNG solution from a bottle covered with aluminium foil to prevent MNNG photolysis, and the solution was replenished every other day.

In week 26, the animals were divided randomly into six groups of 20 rats each. Until the end of the study in week 52, each group was given chow pellets (Nihon Nosan; 60 kcal $day^{-1}$) with or without 10% sodium chloride and subcutaneous (s.c.) injections with or without 0.75 or 1.5 mg $kg^{-1}$ body weight of RA. Group 1, the control group, was given regular chow pellets and s.c. injections of the vehicle (plain olive oil) only; group 2 was given chow pellets containing 10% sodium chloride and s.c. injections of olive oil; group 3 was given chow pellets containing 10% sodium chloride and s.c. injections of 0.75 mg $kg^{-1}$ body weight of RA; group 4 was given chow pellets containing 10% sodium chloride and s.c. injections of 1.5 mg $kg^{-1}$ body weight of RA; group 5 was given regular chow pellets and s.c. injections of 0.75 mg $kg^{-1}$ body weight of RA; and group 6 was given regular chow pellets and s.c. injections of 1.5 mg $kg^{-1}$ body weight of RA. RA (Sigma Chemical Co, St. Louis, MO, USA) was prepared as a suspension in olive oil. Injections were given s.c. in a volume of 1 ml $kg^{-1}$ body weight between 14:00 and 15:00 each day.

### Histological sampling

Animals that survived for more than 50 weeks were included in the effective numbers because the first tumour of the glandular stomach was found in a rat in group 2 that died in week 50. All surviving rats were killed at the end of the experiment in week 52 and examined at autopsy: ten rats from each group were used for cancer study and apoptosis study, and the remaining ten rats from each group for cancer study and bromodeoxyuridine (BrdU)-labelling study. The stomach and other major organs were carefully examined. The stomach was opened along the greater curvature, pinned flat on a cork mat, and fixed with a picric acid–formaldehyde solution for histological examination. The stomach was cut into longitudinal strips 3 mm in width. The specimens were embedded in paraffin, and 5-μm thick serial sections were stained with haematoxylin and eosin for cancer study and with an Apotag kit for apoptosis. The sections were examined without knowledge of the group to which they belonged.

### Definition and classification of gastric cancers

Histologically, adenocarcinomas were defined as lesions in which neoplastic cells had penetrated the muscularis mucosae to invade the submucosa or deeper layers. As previously reported (Tatsuta et al, 1988), adenocarcinomas were classified as very well differentiated, well differentiated, or poorly differentiated.

### Determination of labelling index

The labelling index and also the incidence and number of the gastric cancers were examined in the remaining ten rats from each group in week 52. The labelling index was determined with an immunohistochemical analysis kit for assaying BrdU incorporation (Becton-Dickinson Immunocytometry Systems, Mountain View, CA, USA) (Gratzner et al, 1982; Morstyn et al, 1983). Briefly, non-fasted rats received a s.c. injection of 1 ml $kg^{-1}$ body weight of olive oil (groups 1 and 2), 0.75 mg $kg^{-1}$ body weight of RA (groups 3 and 5), or 1.5 mg $kg^{-1}$ body weight of RA (groups 4 and 6). One hour later, the rats were given an i.p. injection of 20 mg $kg^{-1}$ body weight of BrdU and killed with ether 1 h later. The stomach was fixed in 70% ethanol in 2 N hydrochloric acid for 30 min at room temperature and then in 0.1 M sodium borate to neutralize the acid. The sections were immersed in methanol containing 3% hydrogen peroxide for 30 min and treated with 10% horse serum. Next, the sections were stained with anti-BrdU monoclonal antibody (diluted 1:100) for 2 h at room temperature, and then with biotin-conjugated horse anti-mouse antibody (diluted 1:200) for 30 min. The reaction product was localized with 3,3′-diaminobenzidine tetrahydrochloride. The BrdU-labelled cells were identified by the presence of dark pigment over the nuclei.

The labelling index of the gastric cancers was determined by counting the number of BrdU-labelled and unlabelled cells among 500 cancer cells. The labelling index was expressed as the percentage of BrdU-labelled cells among total number of cancer cells.

### Determination of apoptotic index

The 3′ end-labelling of apoptotic cell DNA was performed with an ApoTag in situ apoptosis detection kit (Oncor, Gaithersburg, MD, USA) (Törmänen et al, 1995) as follows: after dewaxing and dehydration, the sections were incubated with 20 μg $ml^{-1}$ proteinase K (Boehringer Mannheim, Mannheim, Germany) at room temperature for 15 min. Endogenous peroxidase activity was quenched in 2% hydrogen peroxide in phosphate-buffered saline (pH 7.2). Terminal transferase was used to catalyse the addition of digoxigenin-labelled nucleotides to the 3′-hydroxy ends of the fragmented DNA. Antidigoxigenin–peroxidase solution was then applied to the slides. Diaminobenzidine–hydrogen peroxide was used to develop the colour reaction. The specimens were counter-stained lightly with haematoxylin.

The relative number of apoptotic cells in the total tumour cell population was determined by counting the number of apoptotic cells per ×10 high power field. The index was expressed as a percentage of the tumour cells examined.

### Immunohistochemical observation of TGF-α

Immunohistochemistry was performed with the mouse monoclonal antibody AB-2 (Oncogene Science, Cambridge, UK), which is specific for human and rat TGF-α and exhibits no cross-reactivity to epidermal growth factor (Livingstone et al, 1994). Sections were predigested with trypsin for 15 min to expose the antigenic sites before incubation with AB-2 at a dilution of 5:100 overnight at 4°C. After washing with Tris-buffered saline, rabbit anti-mouse serum (Dako, UK) and streptavidin peroxidase complex (Dako) were added at dilutions of 1:333 and 1:400, respectively, for 30 min each before application of diaminobenzidine and counterstaining with haematoxylin. After dehydration in alcohol, the sections were cleared with xylene, and mounted in diphthalate xylene. A positive control section was incubated in each batch to ensure consistency of staining. Two types of negative controls were used: in the first, the primary antibody was replaced by Tris-buffered saline; in the second, specific controls were performed by preincubation of the sections with an excess of the TGF-α peptide PF 008 (Oncogene Science).

TGF-α immunoreactivity was classified as follows: (2+), strong TGF-α expression; (+), weak TGF-α expression; and (–), no TGF-α expression.

### Statistical analysis

The data were analysed with the chi-squared test, Fisher's exact probability test, or one-way analysis of variance with Dunn's multiple comparison (Miller et al, 1966). The data are shown as means ± s.e. Differences were considered significant when the calculated $P$-value was less than 0.05.

### RESULTS

### Incidence, number, histological type, and depth of involvement of gastric cancers

All of the animals were included in the effective numbers because none died before experimental week 50. In week 52, there was no significant difference between the six groups in the body weight of the rats (Table 1).

The incidence, but not the number, of gastric cancers was significantly higher in group 2 (sodium chloride alone; 85%) than in control group 1 (35%) (Table 1). Administration of both sodium chloride and 0.75 (group 3) or 1.5 mg $kg^{-1}$ body weight (group 4) of RA significantly reduced the incidence of gastric cancers

© Cancer Research Campaign 1999

**Table 1** Incidence and number of gastric cancers and body weight in MNNG-treated rats

| Group no. | Treatment[a] | Body weight (g) Week 26 | Body weight (g) Week 52 | Effective no. | No. of rats with gastric cancer (%) | No. of gastric cancers per tumour-bearing rat |
|---|---|---|---|---|---|---|
| 1 | Control | 315 ± 5 | 351 ± 7 | 20 | 7 (35) | 1.0 ± 0.0 |
| 2 | Sodium chloride | 332 ± 4 | 340 ± 7 | 20 | 17 (85)[b] | 1.5 ± 0.2 |
| 3 | Sodium chloride + RA 0.75 mg kg$^{-1}$ | 317 ± 5 | 353 ± 6 | 20 | 8 (40)[c] | 1.1 ± 0.1 |
| 4 | Sodium chloride + RA 1.5 mg kg$^{-1}$ | 323 ± 5 | 351 ± 6 | 20 | 7 (35)[c] | 1.0 ± 0.0 |
| 5 | RA 0.75 mg kg$^{-1}$ | 321 ± 4 | 377 ± 6 | 20 | 10 (50) | 1.0 ± 0.0 |
| 6 | RA 1.5 mg kg$^{-1}$ | 313 ± 5 | 360 ± 11 | 20 | 7 (35) | 1.4 ± 0.2 |

[a]Treatment: group 1, the rats were given regular chow pellets and s.c. injections of 1 ml kg$^{-1}$ of olive oil only every other day after 25 weeks of MNNG treatment; group 2, the rats were given chow pellets containing 10% sodium chloride and s.c. injections of 1 mg kg$^{-1}$ of olive oil only every other day after 25 weeks of MNNG treatment; groups 3 and 4, the rats were given chow pellets containing 10% sodium chloride and s.c. injections of RA (all-*trans*-retinoic acid) at doses of 0.75 and 1.5 mg kg$^{-1}$ body weight, respectively, every other day; groups 5 and 6, the rats were given regular chow pellets and s.c. injections of RA at doses of 0.75 or 1.5 mg kg$^{-1}$ body weight, respectively, every other day after 25 weeks of MNNG treatment. [b]Significantly different from the value for group 1 at $P < 0.01$. [c]Significantly different from the value for group 2 at $P < 0.01$.

**Table 2** Histological type and depth of involvement of gastric cancers in MNNG-treated rats

| Group no. | Treatment[a] | No. of gastric cancers | Histological type (%) Very well differentiated | Histological type (%) Well differentiated | Depth of involvement (%) Submucosal layer | Depth of involvement (%) Muscle layer or deeper |
|---|---|---|---|---|---|---|
| 1 | Control | 7 | 6 (86) | 1 (14) | 6 (86) | 1 (14) |
| 2 | Sodium chloride | 25 | 20 (80) | 5 (20) | 15 (60) | 10 (40) |
| 3 | Sodium chloride + RA 0.75 mg kg$^{-1}$ | 9 | 8 (89) | 1 (11) | 8 (89) | 1 (11) |
| 4 | Sodium chloride + RA 1.5 mg kg$^{-1}$ | 7 | 7 (100) | 0 (0) | 7 (100) | 0 (0) |
| 5 | RA 0.75 mg kg$^{-1}$ | 10 | 10 (100) | 0 (0) | 10 (100) | 0 (0) |
| 6 | RA 1.5 mg kg$^{-1}$ | 10 | 9 (90) | 1 (10) | 10 (100) | 0 (0) |

[a]For an explanation of treatments, see Table 1.

**Table 3** Labelling index, apoptotic index and TGF-α of gastric cancers in MNNG-treated rats

| Group no. | Treatment[a] | Labelling index (%) | Apoptotic index (%) | Immunoreactivity of TGF-α (%) [Apoptotic index, %] No. of cancer examined | (–) | (+) or (2+) |
|---|---|---|---|---|---|---|
| 1 | Control | 24.0 ± 1.0 | 8.6 ± 0.5 | 7 | 7 (100) | 0 (0) |
| 2 | Sodium chloride | 44.2 ± 1.8[b] | 4.2 ± 0.4[b] | 9 | 0 (0) | 9 (100)[f] |
| 3 | Sodium chloride + RA 0.75 mg kg$^{-1}$ | 35.6 ± 1.4[e] | 7.4 ± 0.3[f] | 7 | 4 (57) (8.0 ± 0.0) | 3 (43)[c] (6.7 ± 0.3)[g] |
| 4 | Sodium chloride + RA 1.5 mg kg$^{-1}$ | 32.2 ± 1.6[f] | 8.7 ± 0.5[f] | 7 | 5 (71) (9.4 ± 0.2) | 2 (29)[d] (7.0 ± 0.0)[g] |
| 5 | RA 0.75 mg kg$^{-1}$ | 22.4 ± 1.1 | 9.6 ± 0.5 | 7 | 7 (100) | 0 (0) |
| 6 | RA 1.5 mg kg$^{-1}$ | 23.0 ± 2.0 | 10.6 ± 0.7 | 6 | 6 (100) | 0 (0) |

[a]For an explanation of treatments, see Table 1. [b]Significantly different from the value for group 1 at $P < 0.001$. [c–f]Significantly different from the value for group 2: [c]$P < 0.05$, [d]$P < 0.02$, [e]$P < 0.01$, [f]$P < 0.001$. [g]Significantly different from the value for cancers without TGF-α immunoreactivity at $P < 0.01$.

compared with the results for group 2. Administration of RA alone at either 0.75 (group 5) or 1.5 mg kg$^{-1}$ body weight (group 6) had no significant effect on the incidence or number of gastric cancers when compared with control group 1.

All tumours induced in the glandular stomach were histologically classified as adenocarcinomas (Table 2). There was no significant difference between the six groups in the histological type of the adenocarcinomas. No poorly differentiated cancers were found. Nor was there any significant difference between the six groups in the depth of involvement of the gastric cancers. All of the cancers were found in the antral mucosa and no metastases were found in any of the rats.

### Labelling index, apoptotic index and TGF-α

A diet containing 10% sodium chloride (group 2) significantly increased the labelling index and TGF-α immunoreactivity, but decreased the apoptotic index of gastric cancers compared with control group 1 (Table 3). Administration of both sodium chloride and 0.75 (group 2) or 1.5 mg kg$^{-1}$ body weight (group 3) of RA significantly decreased the labelling index and TGF-α immunoreactivity of the gastric cancers, which were increased by administration of sodium chloride, and increased the apoptotic index, which was reduced by administration of sodium chloride. Administration of RA alone at either 0.75 (group 5) or 1.5 mg kg$^{-1}$ body weight (group 6) had no significant effect on the labelling

*© Cancer Research Campaign 1999*

index, apoptotic index, or TGF-α immunoreactivity of the gastric cancers. In groups 3 and 4, gastric cancers with positive immunoreactivity of TGF-α had a significantly lower apoptotic index than that of cancers without TGF-α immunoreactivity.

### DISCUSSION

The results of the present study show that RA at lower and higher doses attenuates gastric carcinogenesis enhanced by sodium chloride. These findings indicate that RA at 0.75 mg $kg^{-1}$ body weight already provided complete protection against sodium chloride-enhanced gastric carcinogenesis. Therefore, it is still unclear at which concentration RA starts to protect. The mechanism of action of retinoids on tumours appears to be related to their effect on the proliferation and differentiation of the tumour cells themselves (Lippman et al, 1987; Jetten et al, 1990; Lotan, 1994; Liaudet-Coopman et al, 1997). The present study shows that two other mechanisms may be involved in attenuation of gastric carcinogenesis enhanced by sodium chloride.

One is induction of apoptosis by administration of RA. Retinoids have been shown to induce apoptosis in haematological and neuroblastoma models (Delia et al, 1993; Ponzoni et al, 1995). Liaudet-Coopman et al (1997) examined the effect of RA on the growth of the ME-1080 cervical squamous cell carcinoma cell line as a s.c. tumour xenograft in athymic nude mice and found that RA treatment induced apoptosis of the tumour cells and led to a decrease in the tumour growth rate. In a study of the effect of RA and 13-*cis*-retinoic acid on the MCF-7, 2R-75-1 and MDA-MB-231 human breast-cancer cell lines, Toma et al (1997) found that both retinoic acids exerted appreciable dose-dependent growth inhibition, and that the antiproliferative activity of RA was more pronounced than that of 13-*cis*-retinoic acid in all three cell lines tested. Their results also showed that both retinoic acids induced apoptosis in the MCF-7 and MDA-MB-231 cell lines, but not in 2R-75-1. In the present study, combined administration of sodium chloride and RA significantly increased the apoptotic index of the gastric cancers that was reduced by administration of sodium chloride.

A second mechanism that may play a role in the attenuation by RA of gastric carcinogenesis enhanced by sodium chloride is the decrease in TGF-α expression in tumours produced by administration of RA. Livingstone et al (1994) found that the carcinogen MNNG significantly increased the intensity of TGF-α expression in the gastric mucosa and in adenocarcinomas. TGF-α is a 50-amino-acid peptide first isolated from fibroblasts transformed by rodent sarcoma viruses (De Larco and Todaro, 1978) which acts as a ligand for the epidermal growth factor receptor (Derynck, 1986). TGF-α has the ability to induce malignant transformation in some epithelial cells (McGeady et al, 1989; Shankar et al, 1989). Miller et al (1996) found that vitamin A suppresses the proliferation of tracheobronchial epithelial cells in culture by inhibiting expression of the TGF-α gene product. They also noted that similar inhibition of the TGF-α mRNA level by retinol was observed in airway explant cultures and in a cell line immortalized from normal human bronchial epithelial cells. The present study showed that combined administration of sodium chloride and RA significantly decreased the TGF-α immunoreactivity of the gastric cancers that was increased by administration of sodium chloride.

In the present study, however, RA treatment does not influence the underlying tumour incidence in the absence of sodium chloride treatment, nor the apoptotic index in these tumours. Therefore, it is possible that tumours with a lower apoptotic index and higher proliferation rates are selected by sodium chloride treatment, and these are, in turn, selectively inhibited by RA treatment.

The results of the present study showed that administration of RA attenuated gastric carcinogenesis, which was enhanced by the administration of sodium chloride. The findings suggest that both increased apoptosis and decreased expression of TGF-α may be closely related to attenuation of gastric carcinogenesis by RA.

### ACKNOWLEDGEMENT

This work was supported in part by a Grant-in-Aid for the Second-Term Comprehensive 10-year Strategy for Cancer Control from the Ministry of Health and Welfare of Japan.

### REFERENCES

De Larco JE and Todaro GJ (1978) Growth factors from murine sarcoma virus-transformed cells. *Proc Natl Acad Sci USA* **75**: 4001–4005

Delia D, Aiello A, Lombardi L, Pelicci PG, Grignani F, Formelli F, Menard S, Costa A, Veronesi U and Pierotti MA (1993) *N*-(4-hydroxyphenyl)retinamide induces apoptosis of malignant hemopoietic cell lines including those unresponsive to retinoic acid. *Cancer Res* **53**: 6036–6041

Derynck R (1986) Transforming growth factor-alpha: structure and biological activities. *J Cell Biochem* **32**: 293–304

Gratzner HG (1982) Monoclonal antibody to 5-bromo- and 5-iododeoxyuridine: a new reagent for detection of DNA replication. *Science* **218**: 474–475

Jetten AM, Kim JS, Sacks PG, Rearick JI, Lotan D, Hong WK and Lotan R (1990) Suppression of growth and squamous cell differentiation markers in cultured human head and neck squamous carcinoma cells by beta-all-*trans* retinoic acid. *Int J Cancer* **45**: 195–202

Liaudet-Coopman EDE, Berchem GJ and Wellstein A (1997) Inhibition of angiogenesis and induction of apoptosis by retinoic acid in squamous cell carcinoma. *Clin Cancer Res* **3**: 179–184

Lippman SM, Kessler JF and Meyskens Jr FL (1987) Retinoids as preventive and therapeutic anticancer agents. *Cancer Treat Rep* **71**: 391–405 (part I), 493–515 (part II)

Livingstone JI, Filipe MI and Wastell C (1994) Expression of transforming growth factor alpha in experimental gastric carcinogenesis. *Gut* **35**: 604–607

Lotan R (1994) Suppression of squamous cell carcinoma growth and differentiation by retinoids. *Cancer Res* **54**: 1987s–1990s

McGeady ML, Kerby S, Shanker V, Ciardiello F, Salomon D and Seidman M (1989) Infection with a TGF-alpha retroviral vector transforms normal mouse mammary epithelial cells but not normal rat fibroblasts. *Oncogene* **4**: 1375–1385

Miller Jr RG (1966) *Simultaneous Statistical Inference*. McGraw-Hill: New York

Miller LA, Zhao YH and Wu R (1996) Inhibition of TGF-α gene expression by vitamin A in airway epithelium. *J Clin Invest* **97**: 1429–1435

Morstyn G, Hsu SM, Kinsella T, Gratzner H, Russo A and Mitchell JB (1983) Bromodeoxyuridine in tumors and chromosomes detected with monoclonal antibody. *J Clin Invest* **72**: 1844–1850

Ponzoni M, Bocca P, Chiesa V, Decensi A, Pistoia V, Raffaghello L, Rozzo C and Montaldo PG (1995) Differential effects of *N*-(4-hydroxyphenyl)retinamide and retinoic acid on neuroblastoma cells: apoptosis versus differentiation. *Cancer Res* **55**: 853–861

Shankar V, Ciardiello F, Kim N, Derynck R, Liscia DS, Merlo G, Langton BC, Sheer D, Callahan R and Bassin RH (1989) Transformation of an established mouse mammary epithelial cell line following transfection with a human transforming growth factor alpha cDNA. *Carcinogenesis* **2**: 1–11

Shirai T, Fukushima S, Ohshima M, Masuda A and Ito N (1984) Effect of butylated hydroxyanisole, butylated hydroxytoluene, and NaCl on gastric carcinogenesis initiated with *N*-methyl-*N*′-nitro-*N*-nitrosoguanidine in F344 rats. *J Natl Cancer Inst* **72**: 1189–1198

Takahashi M, Kokuba T, Furukawa T, Kurokawa Y and Hayashi Y (1984) Effect of sodium chloride, saccharin, phenobarbital and aspirin on gastric carcinogenesis in rats after initiation with *N*-methyl-*N*′-nitro-*N*-nitrosoguanidine. *Gann* **75**: 494–501

Tatsuta M, Iishi H, Yamamura H, Baba M, Yamamoto R and Taniguchi H (1988) Effect of cimetidine on inhibition by tetragastrin of carcinogenesis induced by *N*-methyl-*N*′-nitro-*N*-nitrosoguanidine in Wistar rats. *Cancer Res* **48**: 1591–1595

© Cancer Research Campaign 1999 

Tatsuta M, Iishi H, Baba M, Yano H, Uehara H and Nakaizumi A (1995) Ornithine decarboxylase inhibitor attenuates NaCl enhancement of gastric carcinogenesis induced by *N*-methyl-*N'*-nitro-*N*-nitrosoguanidine. *Carcinogenesis* **16**: 2107–2110

Tatsuta M, Iishi H, Baba M, Uehara H, Nakaizumi A and Iseki K (1997) Reduction in NaCl-enhanced gastric carcinogenesis in rats fed a high-protein diet. *Cancer Lett* **116**: 247–252

Tatsuta M, Iishi H, Baba M, Hirasawa R, Iseki K, Yano H, Sakai N, Uehara H and Nakaizumi A (1998) Inhibition by transforming growth factor (34–43)-α, a TGF-α antagonist, of gastric carcinogenesis induced by *N*-methyl-*N'*-nitro-*N*-nitrosoguanidine in Wistar rats. *Br J Cancer* **78**: 857–861

Toma S, Isnardi L, Raffo P, Dastoli G, DeFrancisci E, Riccardi L, Palumbo R and Bollag W (1997) Effects of all-*trans*-retinoic acid and 13-*cis*-retinoic acid on breast cancer cell lines: growth inhibition and apoptosis induction. *Int J Cancer* **70**: 619–627

Törmänen U, Eerola A-K, Painio P, Vähäkangas K, Soini Y, Sormunen R, Bloigu R, Lehto V-P and Pääkkö P (1995) Enhanced apoptosis predicts shortened survival in non-small-cell lung carcinoma. *Cancer Res* **55**: 5595–5602

*© Cancer Research Campaign 1999*
